# End-Cycle Sow Carcass Condemnation in a French Slaughterhouse

**DOI:** 10.3389/fvets.2017.00108

**Published:** 2017-07-10

**Authors:** Pierre-Yves Decaudin, Didier Raboisson, Agnès Waret-Szkuta

**Affiliations:** ^1^GMC VETO, ZA Les Gouvernaux, Chabeuil, France; ^2^IHAP, Université de Toulouse, INRA, ENVT, Toulouse, France

**Keywords:** abattoir, sow, condemnation, risk factors, France, data collection

## Abstract

Surveillance at an abattoir allows all animals or carcasses that present a potential public health risk to be withdrawn from the human food chain. Whole-carcass condemnation results in important economic losses, not only for the producer but also for other participants in the meat industry. Access to the personal electronic database of an abattoir in France enabled us to run logistic regression models to investigate the risk factors for whole-carcass condemnation of end-cycle sows in that abattoir. When end-cycle sows that were slaughtered and eviscerated between 22 June 2015 and 8 December 2015 (185 days) were considered (*n* = 19,866), the results highlighted the importance of the total theoretical time off feed, which represents the fasting period from leaving the farm of origin to the time of slaughter (including transportation and waiting time at the abattoir). Each 10-h increase in the theoretical time off feed was associated with a 31% greater likelihood of whole-carcass condemnation [odd ratio (OR) = 1.31, CI 95% (1.27; 1.34)], and a 10 kg increase in carcass weight before refrigeration was associated with a 23% lower likelihood of carcass condemnation [OR = 0.77, CI 95% (0.75; 0.78)]. The results also indicate the importance of the producer group that the farmer belonged to (*P* < 0.01). A relatively small number of variables was available in the actual database to study the relevant risk factors for whole-carcass condemnation associated with or without diseases at the farm of origin. This derives partly from the fact that traceability at the abattoir in France is done per batch rather than individually for pigs; further, limited information is available at the farm level. An investigation of the reasons for whole-carcass condemnation could have been informative; however, it was not feasible in a reasonable timeframe because these data were not saved in a database in a systematic way. Some of the difficulties encountered in this study should soon be alleviated by using the meat inspection information system software for collecting livestock meat inspection data. Implemented recently by the French ministry of agriculture, this new tool should allow for broader perspectives in swine surveillance.

## Introduction

Surveillance at an abattoir allows for all animals or carcasses that present a potential public health risk or that do not satisfy the minimal organoleptic quality requirements to be withdrawn from the human food chain (1). Individual inspection by veterinary services is mandatory (2) and allows inspection data related to animal health to be collected at this level. However, the main difficulty with the use of such data for swine is the lack of data availability and heterogeneity, which depend on the system available in abattoirs both in France and internationally (3).

When high-quality data are routinely collected, they can serve as a basis for epidemiological studies. Studies that identify the risk factors for carcass condemnation based on slaughterhouse data exist for poultry (4, 5), small ruminants (6), cattle (7–9), and swine (10, 11). These studies enabled the geographical origin of the animals (12), time-related parameters (13, 14), the weights of carcasses (3, 15), and seasonality (11, 16, 17) to be identified as having a potential influence on whole-carcass condemnation. One influence could be stress during transport periods, which is associated with diminished carcass quality (17). Nonetheless, information on this topic remains scarce, and when whole-carcass condemnation occurs, it results in important economic losses for both the producer and the other participants in the meat industry.

In 2013, 23.5 million pigs were slaughtered in France, producing 2.199 million tons of pork carcass weight equivalents. France is the third largest pork producer in the EU, behind Germany and Spain (18). According to the last agricultural census in 2010, France had 13.8 million pigs, with 1.1 million sows present throughout the country. Most French pig production (91%) is carried out by farmers who are members of one of the 42 producer groups in the country that also have dominant roles in Denmark, Finland, Sweden, and Malta (19). The primary function of the producer groups is twofold: to disseminate technical information and genetic progress, to improve the competitiveness of farms, to better meet the needs of the downstream industries, and to ensure the marketing of pigs of their members. The dominant pig farming system in France includes the farrowing and fattening of the animals. The French sow herd is concentrated in 5,700 farms with 50 or more sows, with an average size of 190 sows in 2010. The number of farms with less than 100 sows has greatly decreased, but they remain numerous (45% of the farms in 2010) (18).

Sows are managed per batch, with an average annual turn-over of 40% after weaning 28.7 piglets over their reproductive lifespan (18). Sows can be considered unfit for service for different reasons, but it is most often because of diminishing reproductive performance. Managing end-cycle sows may be difficult because their economic value must be optimized to ensure sufficient fattening without spending too much on feed to provide an acceptable return on the investment and because departure from the abattoir must be arranged with animals from other farmers because the low number of sows per batch cannot fill a transport truck.

The aim of this study was to investigate the potential risk factors for whole-carcass condemnation by reviewing 1 year of meat inspection data from a French abattoir. We focused on end-cycle sows because this site was one of the largest French slaughterhouses for this category of animals.

## Materials and Methods

### Data

The main study population consisted of end-cycle sows slaughtered between 22 June 2015 and 08 December 2015 (dataset 1, 185 days, *n* = 22,609), for which information on the status of evisceration (yes/no) was available. This criterion was selected to perform the main analysis and investigate carcass weight as a potential risk factor for condemnation. Therefore, only sows with a known eviscerated carcass weight before refrigeration were kept in dataset 1 (*n* = 19,866). It included end-cycle sows with carcasses that had been considered healthy at the time of veterinary inspection (*n* = 19,517) and end-cycle sows that were eviscerated but wholly condemned (*n* = 349), with up to three reasons for condemnation considered. The animals were provided by 22 producer groups from 47 departments (France is divided into 97 departments) and were transported by 17 different transport operators. The database included the date and hour of the last meal the sow ate, the date and hour of sow departure from the farm, the date and hour of arrival at the abattoir, and the date and hour of slaughter.

A second dataset includes all end-cycle sows that were (i) slaughtered between 14 January 2015 and 6 December 2015 and were (ii) wholly condemned or not condemned (dataset 2, *n* = 44,426). It included dataset 1, but evisceration status was not completely available for all sows in dataset 2. The weight after evisceration was not available in dataset 2.

Several variables were generated. The variable duration of transport resulted from the difference between the date and hour of arrival at the abattoir and the date and hour of departure from the farm. The variable waiting time at the abattoir (*wta*) resulted from the difference between the date and hour of arrival at the abattoir and the date and hour of slaughter. Three variables related to fasting periods were defined: the time off feed before arrival at the abattoir (the difference between the date and hour of arrival at the abattoir and the date and hour of the last meal of the sow), the total theoretical time off feed [obtained by the difference between the date and hour of slaughter and the date and hour of the initiation of the fasting period at the farm, theatrical time off feed (*Ttof*)], and the fasting period at the farm (the difference between the date and time of departure of the sow from the farm and the date and time of the start of the fasting period at the farm, *fpf*).

### Modeling Methods and Software

The variables of interest of dataset 1 and dataset 2 were compared with a Wilcoxon test to detect any difference in the two datasets. Similarly, a Wilcoxon non-parametric test was performed to compare the characteristics of sows that were or were not condemned. The condemnation rate per department was mapped with ArcGis 10.4.

Then, univariate logistic models for the outcome variable “carcass condemnation” (0 or 1) were run for each of the explanatory variables. The explanatory variables were either categorical, including producer group (21 classes), department (27 classes), and transport operator (17 classes), or quantitative and continuous, including carcass weight (*W*), *wta*, and the three fasting-period variables. The level of significance for the statistical tests was set at 0.05.

Finally, a multivariable logistic model was used with whole-carcass condemnation as the outcome variable (0 or 1) using a step-wise ascendant procedure. Akaike information criterion (AIC) and likelihood comparison tests were used to assess the goodness of fit of the different models. The variables department and producer group were forced (alternatively) as random or fixed effects. The coefficients of all others fixed variables did not differ between the fixed-effect and mixed-effect models (i.e., between regressions with the department or producer group as a fixed or random effect). Consequently, only the fixed-effects models were kept and presented here. Interactions between the different variables were tested. Statistical analyses were performed with R version 3.2.3 (20).

## Results

### Descriptive Analysis

**Figure 1:**
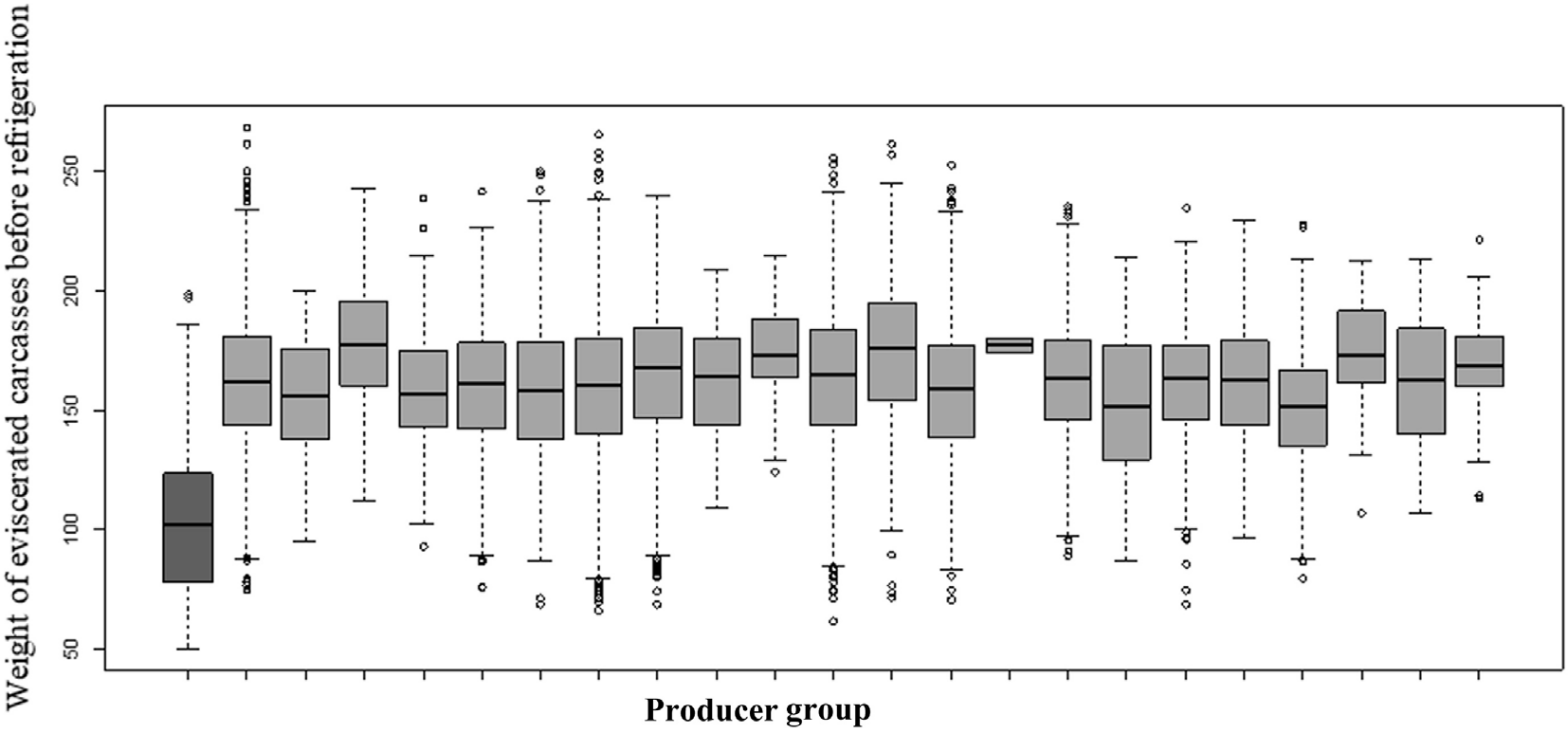
Distribution per producer group of the weight of the eviscerated carcasses before refrigeration (*W*). The first producer group, shown on the left of the figure, was discarded from the analysis to avoid introducing bias (353 sows, with 3 condemned).

**Figure 2:**
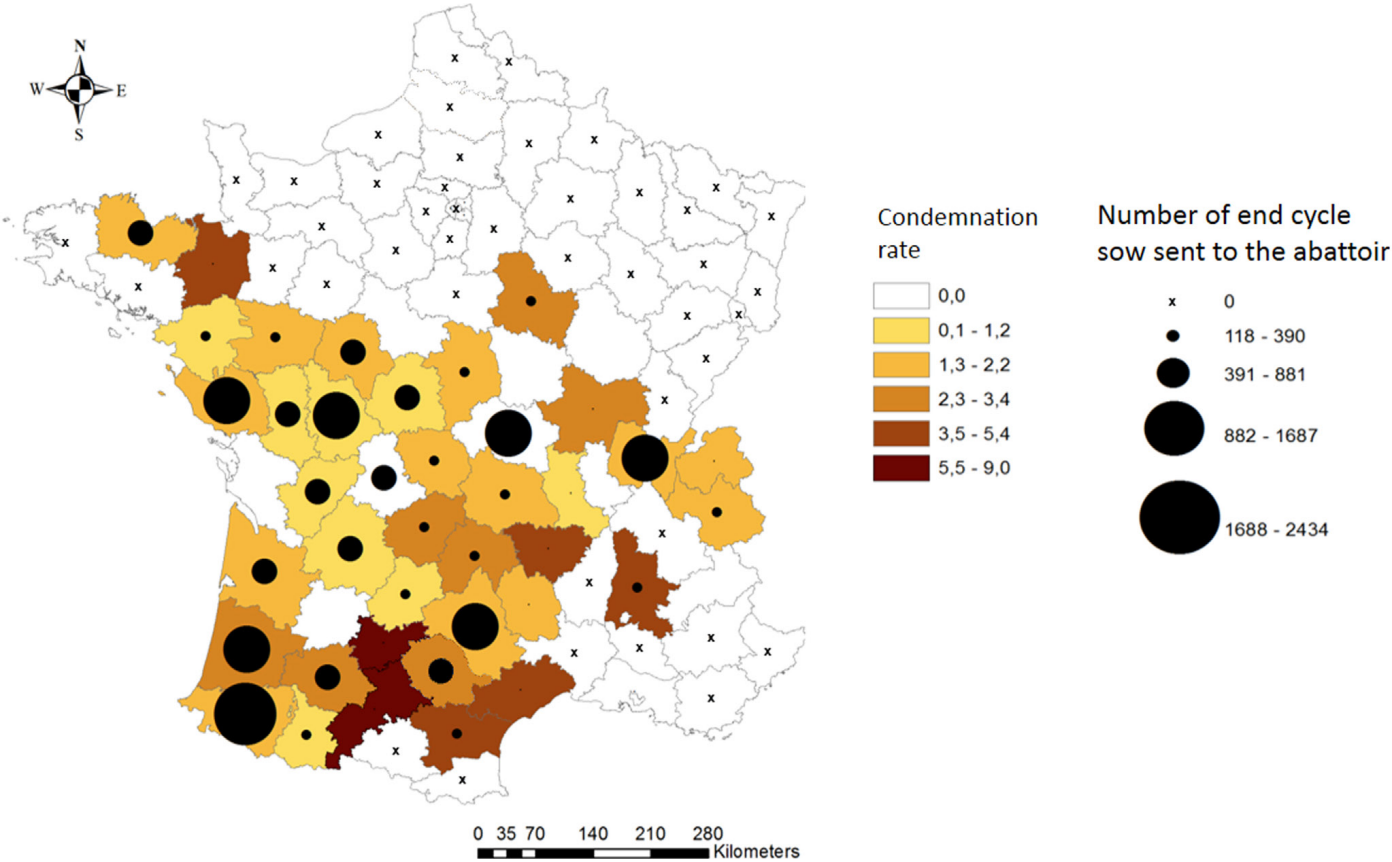
Number of end-cycle sows sent to the abattoir and the condemnation rate per department (27 departments).

Descriptive statistics are provided for dataset 1 since it was the focus of the main analysis (Table 1; Figure 1). No significant changes were observed in the descriptive statistics for dataset 2. The percentage of total condemnation was of 1.76% (dataset 1). The median *W* among producer groups was uniformly distributed and ranged between 150 and 170 kg. The producer group with a median *W* of 102 kg was not included in the final analysis to avoid introducing bias. The difference in the median *W* between end-cycle sows that were not condemned (162.2 kg) and those that were wholly condemned (139.9 kg) was significant (*P* < 2.2 × 10^−16^). No particular spatial pattern in departmental condemnation rates was observed (Figure 2). The analysis of the reasons for carcass condemnation for end-cycle sows with *W* < 140 kg (*n* = 151) showed that the first three reasons for whole-carcass condemnation were non-specific abscesses in multiple locations, arthritis, and pleurisy.

**Table 1:** Descriptions of the continuous variables included in the analysis, including minimum, maximum, median, mean, and SD values (dataset 1).

	Minimum	Maximum	Median	Mean	SD
Carcass weight before refrigeration (*W*, kg)	61.3	268.5	161.9	160.9	28.5
Transport duration (h)	1	35.5	9.0	9.4	4.7
Waiting time at abattoir (*wta*, h)	1	48.6	16.2	15.7	5.8
Total theoretical time off feed (*Ttof*, h)	13.9	75.1	40.5	40.7	6.6
Time off feed before arrival at abattoir (h)	2.5	67.0	23.5	25.0	7.0
Fasting period at the farm (*fpf*, h)	0	50.5	14.5	15.5	5.7

### Results of Logistic Regressions

The results of the univariate models did not show significant associations between carcass condemnation and the duration of transport or the time off feed before arriving at the abattoir. Month and season were not significantly associated with carcass condemnation. The non-adjusted odds ratio for carcass condemnation was 0.78 for each extra 10 kg of *W*. The odds of carcass condemnation were 30, 23, and 22% higher [ORs = 1.30 (95% CI = 1.22–1.37), 1.23 (95% CI = 1.14–1.32), and 1.22 (95% CI = 1.13–1.31)] for each extra 10 h of *Ttof, wta*, and *fpf*, respectively.

**Figure 3:**
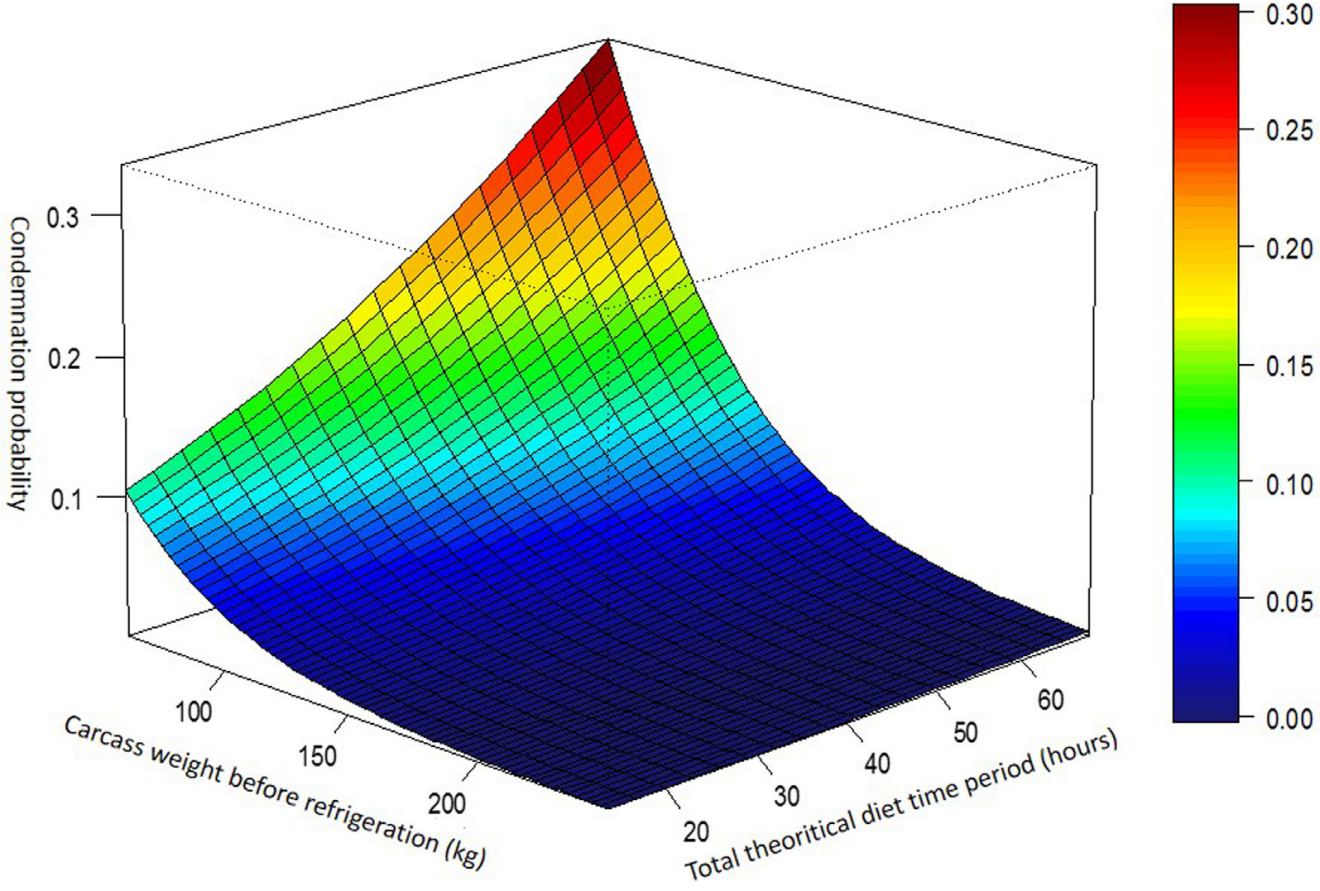
Graph of the probability of whole-carcass condemnation depending on carcass weight (*W*) and total theoretical time off feed.

In different variable combinations, confounding effects were found between the variables producer group and department. These two variables were highly correlated and were included alternatively in the models. The best logistic regression model (Table 2) appeared to be the one that included the variables *W*, producer group, and *Ttof* (model 1), although the model that included department instead of producer group was also robust (model 2). Each additional 10 h of *Ttof* was associated with a 31% greater likelihood of whole-carcass condemnation [OR = 1.31, CI 95% (1.23; 1.38)], and a 10 kg increase in *W* was associated with a 23% lesser likelihood of carcass condemnation [OR = 0.77, CI 95% (0.75; 0.79)]. Significant differences in carcass condemnation were observed between the different producer groups. The theoretical curve representing the variation in the probability of end-cycle sow whole-carcass condemnation depending on *W* and *Ttof* and adjusted for producer group (model 1) is presented in Figure 3.

**Table 2:** Results of the two different best logistic regression models for the outcome variable carcass condemnation, including three variables.

			β_i_	SE	Odd ratio	95% CI	*P*-value	Akaike information criterion
Dataset 1	Model 1^a^	Intercept	−9.5 × 10^−1^	4.6 × 10^−1^				3,313
		+*W*/10 kg	−0.26	1.9 × 10^−2^	0.77	0.75–0.79	<2.0 × 10^−16^	
		+*Ttof*/10 h	0.27	7.7 × 10^−2^	1.31	1.23–1.38	5.4 × 10^−4^	

	Model 2^b^	Intercept	−9.9 × 10^−1^	4.5 × 10^−1^				3,326
		+*W*/10 kg	−0.26	1.9 × 10^−2^	0.77	0.75–0.78	<2.0 × 10^−16^	
		+*Ttof*/10 h	0.24	7.8 × 10^−2^	1.27	1.19–1.35	1.9 × 10^−3^	

Dataset 2	Model 3^c^	Intercept	−3.9	1.9 × 10^−1^				11,472
		+*Ttof*/10 h	0.14	3.9 × 10^−2^	1.15	1.11–1.19	2.8 × 10^−4^	

	Model 4^d^	Intercept	−3.9	1.9 × 10^−1^				11,453
		+*Ttof*/10 h	0.082	4.1 × 10^−2^	1.08	1.04–1.13	4.8 × 10^−2^	

*^a,c^Variable producer group is included but is not reported here (21 classes)*.

*^b,d^Variable department is included but is not reported here (27 classes)*.

*W, carcass weight; Ttof, total theoretical time off feed*.

When the extended dataset 2 was considered, similar results were found, except for those related to *W*, which was not yet available at the time of analysis (models 3 and 4). No interactions were found.

## Discussion

### Risk for Sow Carcass Condemnation

Light carcass weight at slaughter (*W*) appeared to be the main risk factor for the total condemnation of an end-cycle sow at the abattoir in our study. This could be explained by the fact that a diseased end-cycle sow will have reduced weight gain because of lower ingestion rates, although its metabolic needs are increased (21, 22). Alternatively, or simultaneously, a low-weight sow is probably more vulnerable outside the farm, for example, when the hierarchy is settled during transport and is thus at a greater risk of being injured. An increase in the theoretical total off feed period (*Ttof*), representing the delay between the last meal of the sow at the farm and the time of slaughter, also appears to increase the risk of total condemnation of the carcass. This could be interpreted concomitantly with increased stress and possible aggressiveness when *Ttof* increases, possibly impacting meat quality (17, 23, 24). The significant differences found between the producer groups did not appear to be associated with their localization, which can recover contiguous departments, although the two variables are confounded. The variable producer group may also include differences in technical levels or some specifications of the producer groups. The variable month or season was not significant in our analysis, which is consistent with previous results in end-cycle sows (11). Seasonal influence on mortality during transport has yet not been regularly reported (25, 26).

### Improvement of Data Recording in Slaughterhouses

A relatively small number of variables was available in the actual database to study the relevant risk factors for whole-carcass condemnation associated with diseases at the farm of origin. This derives partly from the facts that traceability at an abattoir in France is per batch of pigs rather per than individually and that limited slaughterhouse information is available at the farm level. It would have been interesting to have data on sow parity, age, or reason for withdrawal at the farm level. In addition, it could have been useful to have information related to the sanitary status of the farm of origin, mortality rate at the farm, and the type of farm (farrowing or farrowing to weaning or farrowing to fattening). The structure of the dataset did not allow to identify a subpopulation to build the model and another one to validate the proposed model, since it would have introduced biases related for instance to the spatial distribution of the department of origin and to the different times before slaughter.

An investigation of the reasons for whole-carcass condemnation could have been informative, but this was not feasible in a reasonable timeframe because the data were not saved in a database in a systematic way. A number of possible combinations of reasons for condemnation are likely because up to three different reasons can be cited in different orders, although one or two reasons may occasionally be sufficient. However, the first analysis on the subset of sows weighing less than 140 kg suggests that the reasons for condemnation in the abattoir studied here are similar to the major reasons found for swine carcass condemnation in all French abattoirs (27) but are different from those in Portugal; for example, see Garcia-Diez and Coelho (11). This is probably due to different geographical locations, variable climatic conditions, different rearing systems at the farm level, and different sanitary statuses of the animals (10). Abscesses, arthritis, and pleurisy are lesions that develop over moderate to long time periods and that do not appear to be compatible with time spent at the abattoir. Refining this analysis to include all end-cycle sows available in the database could possibly allow a better interpretation of the time-related risk factors. Indeed, depending on the major lesions recorded and the delay in their appearance, the timeframe, or component of the production chain to focus on could be more appropriately identified (farm, transport, or abattoir) to optimize resource allocation and gain useful and reliable information.

The results presented here constitute a foundation for discussions among the different actors in the pork production chain, with some descriptive results that differ from those that were expected in relation to quality recommendations or legal frameworks. Waiting times at the abattoir appeared to be excessive in some cases, or the fasting periods were too long. One possible explanation is that the recorded information does not allow a precise trace back to the transport of end-cycle sows. Indeed, because of the size of the production farms in France, trucks often transport end-cycle sows that belong to different farms that can be relatively far from one another, and transport can include stops that are not mentioned in the records. These circumstances can result in a time off feed that is much longer than the records show. In addition, a retranscription of data errors or mistakes in paper records may occur. However, proper reporting of elapsed times and rigorous paper work were identified as areas of potential progression in the quality assurance standards. The weights of carcasses at slaughter also revealed that the presence of animals less than 120 kg in the chain of production should be investigated further.

Some of the difficulties encountered in this study should soon be alleviated by the national meat inspection information system software (SI2A in French) for livestock meat inspection data collection. Implemented on 1 January 2015 by the French ministry of agriculture in the 273 French meat abattoirs, this new tool should provide broader perspectives for swine surveillance (28) because the data will be uniformly entered and centralized. However, currently, the first objective of the system is to improve administrative veterinary notifications, so, information is only saved for carcasses with anomalies, thus preventing a simple re-running of our type of analysis. This should be considered in a second step in the advancement of the software.

## Author Contributions

P-YD co-drafted the paper and performed the statistical analysis, including the interpretation of the results. DR contributed to the conception of the analysis, critically revised the paper, and helped in the interpretation of results. AW-S designed the study, acquired the data, critically revised the paper, and helped in the interpretation of the analysis. All authors approved the final version of the paper.

## Conflict of Interest Statement

The authors declare that the research was conducted in the absence of any commercial or financial relationships that could be construed as a potential conflict of interest.
